# The Value of Animal Experimentation

**Published:** 1896-11

**Authors:** 


### The Value of Animal Experimentation.

At the close of the Semi-Centennial exercises, Lord Playfair, who was present, was asked to speak.

Lord Playfair expressad special interest in the celebration now in progress, partly due to the fact that he himself in the course of his life's work had made numerous experiments relating to means and methods of anesthesia.

On every occasion of this sort the name of Sir James Simpson mnst be mentioned with gratitude. The disinterested enthusiasm with which Dr. Simpson worked regardless even of actual danger to himself, was most praiseworthy.

Lord Playfair told an amusing story of an experiment which he was about to conduct with Sir James Simpson in the direction of a supposed new anesthetic method. Sir James came to him one day and told him that he was disgusted with chloroform and would thank him very much for the discovery of a satisfactory substitute. Lord Playfair a few days later announced to him that he had made the required discovery. The material that he intended to use was bi-bromide of etholene. Sir James Simpson smelt the compound, and forthwith said that it was the very thing wanted. He was very anxious to repair immediately to Lord Playfair's private room and experiment upon himself.

Lord Playfair was unwilling that the experiment should take place before further trial, and finally induced Sir James to have the anesthetic tried on some rabbits first. The rabbits were accordingly treated, and were put away to await developments.

On the next day Dr. Simpson appeared at Lord Playfair's laboratory, propped himself up with two chairs, and asked Lord Playfair for the solution. Lady Simpson, who was present, advised her husband to see how the rabits had fared under the treatment before he applied it to himself.

"When the attendant came in," continued Lord Playfair, "we saw him holding by the ears two rabbits—perfectly dead."

The story is certainly a striking instance of the value of animal experimentation.

Although an Englishman, Lord Playfair felt that the credit for the discovery of anesthesia belonged to the United States and to Dr. Morton, who first made the world realize its value. —*Boston M. & S. Journal.*

